# Drug‐Use Safety Enhancement Model—Theory and Application

**DOI:** 10.1002/puh2.70016

**Published:** 2024-12-21

**Authors:** Edward T. Dunbar, Ania Bartkowiak, Alison L. Brennan

**Affiliations:** ^1^ Department of Counseling Montana State University Bozeman Montana USA; ^2^ Department of Human Development and Community Health Montana State University Bozeman Montana USA

**Keywords:** drug use, drug‐use safety, drug‐use stigma, harm reduction

## Abstract

Harm reduction strategies mitigate the adverse effects of problematic drug use through overdose prevention, disease transmission reduction, and improved access to treatment resources. However, educational resources for safe drug use remain sparse and are predominantly focused on abstinence‐based approaches. This manuscript introduces the Drug‐Use Safety Enhancement Model (DUSEM), a comprehensive framework designed to foster informed and healthy relationships with drugs. Grounded in interdisciplinary research, DUSEM encompasses eight domains: knowledge, motivation, set (mindset), setting, dose, administration, recovery, and evaluation. Each domain offers practical strategies for educators, professionals, and drug consumers to enhance drug‐use safety. For the purpose of this analysis, “drugs” include all psychoactive substances regardless of whether they are legal in the United States. Our approach is rooted in the ethical, legal, and cultural practices common in the United States, particularly those related to best practices for providing care to people who use substances. The American context shaped how we understand and talk about drug use, which reflects the American reality of clinical practice in the fields of mental health and addictions. From the perspective of evaluation of the traditional, American conceptualization of the issues around drug use, our model's development acknowledges the need for a shift from outdated abstinence‐focused paradigms toward empowering individuals with informed practices for safer drug use. While serving as an educational guidepost, the model underscores the necessity for further research to refine its application, efficacy, and curriculum development. Helping professionals, such as teachers, counselors, social workers, and psychologists, can use this model in various educational, clinical, or institutional settings to help their audiences explore their own relationships with drugs. Ultimately, DUSEM aims to destigmatize drug use, foster healthier relationships with drugs, and improve safety for consumers.

## 1 Introduction

Harm reduction, a community‐driven public health strategy, helps mitigate the adverse effects of drug use by preventing overdoses, reducing the transmission of infectious diseases, and facilitating access to treatment resources for people with problematic drug use [1]. In recent years, harm reduction strategies, such as needle exchanges, overdose prevention kits, and safe injection sites, have gained increasing popularity. Despite their proven effectiveness in minimizing the harms associated with high‐risk drug use [2], unbiased educational resources for drug consumers remain limited [3], and guidelines for safe drug use are poorly defined. Drug‐use educational resources remain skewed toward addiction treatment and abstinence from illicit drugs, which has resulted in a dearth of information and strategies addressing drug‐use safety. The purpose of this article is to outline a comprehensive educational framework for enhancing drug‐use safety that helping professionals can use in various settings. This work aims to fill the gap between harm reduction concepts and practical applications in clinical, educational, and institutional settings.

Even though the use of psychoactive substances dates back thousands of years, our knowledge and resources for promoting safe drug use remain inadequate [4]. Today, over 90% of the global population consumes psychoactive substances, including legal ones like caffeine [5], yet our knowledge about drugs is inaccurate and often lacks a scientific foundation [3, 4]. Throughout history, humans have utilized psychoactive drugs for various purposes, such as pain relief, enhanced energy, spiritual exploration, and improved social interactions [6, 7]. In contemporary Western society, motivations for drug‐use mirror those of our ancestors, encompassing recreational, stress management, performance enhancement, and the alleviation of co‐occurring mental and physical health symptoms [8].

American drug consumption intersects with our political and cultural attitudes and policies, which is reflected in changes following the Civil Rights Act (1964) and subsequent Controlled Substances Act in 1970. Over time, our approaches to drug production, distribution, and consumption have undergone advancements. These advancements necessitate similar advancements in education, attitudes, and policies.

Over time, agriculture, science, and processing developments have yielded potent, easily accessible drugs. However, these advancements have outpaced our societies’ attitudes, education, and social policies about drugs and drug use. As a result, drug‐use stigmas and biases are ingrained in our healthcare, education, and social policies [9]. Due to these stigmas and biases, education and treatment for drug‐related issues often rely on outdated and ineffective abstinence‐based treatment approaches, which further reinforce stigmas about drug use [9]. This negative feedback loop has led to a dearth of resources for education, prevention, and treatment of drug use that emphasizes developing healthy relationships with drugs. Rather, many of our resources continue to belabor outdated abstinence‐based approaches that are often incongruent with consumers’ motivations. Although abstinence‐based approaches may work for some people, mounting evidence supports individualized harm reduction approaches tailored toward individuals’ needs, development, goals, and motivations [10, 11, 12, 13, 14].

For example, in the 2022 results of the National Survey on Drug Use and Health, 67.4% of US adults had consumed alcohol in the past year, 52.9% had consumed alcohol in the past 30 days, 23.5% had engaged in binge drinking in the past 30 days, and 11.2% reported past‐year alcohol use disorder [15]. Promoting abstinence from alcohol would be incongruent for most US adults, but a harm reduction approach might be more sensible and acceptable to consumers. In fact, SAMHSA's main informational webpage about alcohol focuses on defining and providing examples of a standard drink, signs of excessive drinking, and ways to set and track daily and weekly limits on an individually determined basis [16].

Although most people who use drugs do so without developing problems such as addiction [4, 17, 18, 19, 20], resources for teaching people about drug‐use safety are virtually non‐existent. Additionally, the context of the providers’/educators’ cultural identity and intersections of identity will affect how different audiences receive and integrate the psychoeducation offered by the providers.

Consideration of culture is essential among providers who support patients who use drugs and those dealing with addiction, and providers must continually adapt to changing culture by learning and implementing different cultural norms and values while practicing humility [21]. Ancis [22] provided an update to the expectations for addiction counselors, summarizing and emphasizing adaptations necessary to ensure culturally responsive and relevant treatment of addiction. Moving forward, three critical expectations for addiction counselors are that they will commit to

1. Incorporating cultural values in existing settings.
2. Culturally adapting evidence‐based interventions.
3. Implementing novel strategies tailored to cultural norms [22].

As readers review different portions of the Drug‐Use Safety Enhancement Model (DUSEM) described in the sections that follow, we encourage them to reflect on these three tenets in conceptualization and implementation of the model.

## 2 Model Development

The DUSEM is a pragmatic and interdisciplinary model applicable across educational, research, and clinical settings aimed at fostering individuals’ healthy, informed relationships with drugs. This model draws upon research from diverse fields, including addiction studies, medicine, nursing, psychology, and public health. The model's primary objective is to equip individuals who use drugs with practical skills and strategies to bolster their drug‐use safety practices. Consequently, this model is a resource for professionals working in the field to educate and guide their clients about principles and practices for enhancing drug‐use safety. The model was developed to be accessed by both clinical professionals and the broader public who use drugs or who are interested in learning how to use drugs safely.

### 2.1 The Model

The DUSEM encompasses eight distinct domains that serve as a comprehensive framework for guiding educators, helping professionals, and drug consumers in enhancing drug‐use safety. These domains can be utilized either sequentially or in any order deemed appropriate. Moreover, the model may be tailored to align with individuals, families, groups, and classroom settings’ diverse educational levels, motivations, and learning preferences.

The DUSEM resembles holistic models of human well‐being, as its domains offer multidimensional strategies to enhance drug‐use safety. Initially, these domains can be utilized to educate and counsel drug consumers on the fundamental principles of drug‐use safety. Additionally, the interactions between these domains can also enhance drug‐use safety. For instance, when someone explores the knowledge domain, they may become aware of their own misconceptions related to the drug they are using, which, in turn, can heighten their understanding of the drug's impact within the set (mindset) domain and lead to behavioral changes in the dose and administration domains.

The following overview of the core concepts of the DUSEM can be used to help educate, train, and research drug‐use safety. Although not an exhaustive list, each domain includes examples of practical strategies that drug consumers and professionals could use to enhance their drug‐use safety.

#### 2.1.1 Domain 1: Knowledge

The first domain, termed “knowledge,” pertains to an individual's acquired information relevant to the drugs they intend to use. Drug use often begins during adolescence and some drug use is developmentally normative, though there are important distinctions among specific substance use trajectories (for a review, see [19]). Notably, adolescents often acquire knowledge about drugs through direct consumption or vicariously through friends or media portrayals rather than through formal education [23, 24]. Simply put, adolescents often learn about drug use by using drugs rather than by learning about drug‐use safety beforehand. This experiential approach may result in unhealthy relationships with drugs as adolescents transition into adulthood, as drug use trends in adolescence may predict adult use trends [25]. Although most adolescents who use drugs do not develop substance use disorders in adulthood, the significance of early experiences with substance use must not be discounted [20]. Additionally, as discussed, advancements in drug‐use potency increase the risks of this trial‐and‐error approach and can lead to accidental overdoses and other high‐risk behaviors.

The knowledge domain of this model can be used to educate individuals who use or intend to use drugs about the benefits, side effects, and interactions of different substances. Furthermore, it can help dispel misconceptions and myths related to drug use. For instance, when working with an adult client interested in using cannabis products, a helping professional can utilize this domain to assess the client's existing knowledge about how cannabis works, its potential benefits, and its side effects. In this example, this process also allows for the exploration and correction of common myths associated with cannabis, which may have been obtained from unscientific sources such as peers, family, or the internet.

##### 2.1.1.1 Practical Applications

Safety enhancement methods from the knowledge domain may contain educational content on the benefits and side effects of various drug types, drug interactions, common drug myths, neurotransmission, and acute/chronic effects and could employ resources such as quizzes, websites, apps, and articles to promote ongoing drug education.

#### 2.1.2 Domain 2: Motivation

The motivation domain in the DUSEM considers why someone uses drugs at a given moment. Motivation for drug use in this domain is conceptualized along a continuum from enhancing pleasant experiences to using drugs to escape from negative cognitive, physical, emotional, or social states. This approach builds on the previous research on drug‐use motivations [26] by presenting drug‐use motivation as a spectrum ranging from escape to enhancement, which may aid in its applicability in clinical and educational settings.

The motivation domain can be used to help drug consumers explore their motivation for use at a given time to determine any risks associated with their potential drug use. For example, people with mental health disorders have increased risks of problematic drug use [8]. This domain could help drug consumers determine if they are contemplating drug use or engaging in drug use to escape from feelings of anxiety and depression, which may indicate high‐risk drug use [19]. Examples of enhancing experiences may include recreation, social drug use such as concerts or events, spiritual enhancement such as religious ceremonies, or seeking insights through altering consciousness by using drugs.

##### 2.1.2.1 Practical Applications

Safety enhancement methods within the motivation domain may include visual representations of the spectrum of drug‐use motivations from escape to enhancement, journaling activities addressing drug‐use intentions, and personalized identification of factors associated with escape and enhanced motivational states.

#### 2.1.3 Domain 3: Set (Mindset)

The third domain of the DUSEM is called set or mindset. This domain refers to the states and traits that an individual brings to a specific drug‐use experience [27]. Traits may be personality characteristics and enduring belief systems, whereas states refer to temporary ways of being, such as mood, anxiety, and stress levels.

State characteristics also include chronic and acute mental health issues, which can influence drug‐use experiences. People with unaddressed mental health issues may be vulnerable to developing problematic relationships with drugs. Because co‐occurring issues are common with problematic drug use [8], they are emphasized in the set domain as a point of counseling, education, or prevention. For example, helping professionals educate from the set domain can teach their clients about the relationships between mental health and drug use. It can additionally help clients determine mental health states that may be conducive to safe drug use and mental health states that could lead to risky drug use.

Although personality traits, like neuroticism and sensation‐seeking, have been linked to drug use in previous studies [28, 29], their efficacy as predictors of problematic drug use remains uncertain. Studies suggest that neuroticism and sensation‐seeking may be indicative of illegal drug use. However, further research is required to ascertain whether these traits are reliable indicators of the severity of drug use or the progression to problematic use [28, 29]. Nonetheless, individuals exhibiting high levels of impulsivity or enduring patterns of emotional instability might be vulnerable to engaging in risky drug behaviors.

Expectations are included in the set domain as they often predict the outcomes of drug‐use events. The effects of drugs are more correlated with what people think the effects of the drug will be rather than the drugs’ actual effects [27]. For example, people who believe stimulants or alcohol cause aggression and violence are more likely to become aggressive and violent when they use alcohol or stimulants. The set domain can be used to explore a person's beliefs about a drug's effect, which may also relate to the knowledge domain. For example, a person planning to use alcohol may expect it to decrease anxiety and increase sociability. Although this may be true, exploring these expectations may provide a helping professional the opportunity to educate, via the knowledge domain, this person about the interplay between anxiety and alcohol in which alcohol may decrease acute anxiety but raise chronic anxiety levels [30].

##### 2.1.3.1 Practical Applications

Safety enhancement methods from the set domain include journaling, mood/anxiety tracker apps and worksheets, biofeedback, stress reduction training, psychoeducation about drug use and mental health, drug‐use family mapping, and explorations of beliefs and expectations about drug use.

#### 2.1.4 Domain 4: Setting

The fourth domain of the DUSEM is termed setting and refers to the environment in which the drug is taken. The setting domain includes the physical, cultural, and social, environments where drug use occurs [27, 31]. Prior research on setting has focused on the environment in which drugs were taken and the effects experienced. However, the setting domain in this model emphasizes setting throughout the drug‐use experience, including the legal status of the drug and its use that varies based on geography.

This domain considers setting throughout the drug‐use experience to enhance drug‐use safety. This longitudinal perspective is considered along a continuum of behaviors relating to drug use. This includes obtaining, preparing, ingesting, onset effects, metabolization, and elimination. The role of setting and safety can be emphasized to help people who use drugs consider behavioral steps throughout their drug‐using experience. For example, this model includes metabolization and elimination in settings because some people who use drugs may travel between locations after they believe they are no longer experiencing a drug's effects, which may be an increased risk factor for clients in rural settings [32].

##### 2.1.4.1 Practical Applications

Safety enhancement methods from the setting domain may include methods such as planning drug use around other people, obtaining safe transportation to and from a drug‐use event, identifying safe people to use drugs around, and safety methods relevant to various settings. For example, common safety enhancement methods that would be categorized in the setting domain of this model are avoiding leaving beverages unattended and using a designated sober driver. In research conducted by Linden‐Carmichael and colleagues [33], some setting‐related protective behavioral strategies were associated with reduced likelihood of experiencing acute harmful drinking‐related consequences among college students who engaged in high‐intensity drinking, and the researchers suggested that interventions for heavy drinking include a focus on planning strategies to minimize harm.

Additionally, the people who are present in the direct social environment may be of critical importance if an emergency, such as an overdose, occurs. One potential application that combines the knowledge and setting domains would be universal training in basic bystander response skills for substance use crises, including identification of overdose signs, key supportive actions (e.g., putting a person into the recovery position), and awareness of legal protections directly related to drug use and handling of crisis situations (i.e., Good Samaritan laws).

#### 2.1.5 Domain 5: Dose

The fifth domain of the DUSEM is dose, which refers to the amount of active and inactive ingredients in a drug needed to achieve desired results [34]. This domain considers drug content issues, including sourcing, potency, and purity. It can be used to help people who use drugs apply practical measures to legal and illicit drugs to promote drug‐use safety.

Drug consumers and helping professionals addressing the dose domain can consider promoting safety during the sourcing or obtaining of a drug by considering the locations the drug will be sourced from its reliability, and any possible risky behaviors that may arise during the sourcing process. Sourcing may also entail discussions about how drugs are manufactured, processed, and delivered as legal drugs, such as pharmaceutical drugs, have regulated ingredients, processes, and purity standards, whereas illicit drugs do not.

Potency can also be considered efficient because more potent drug doses require smaller amounts than less potent ones. Because of their unregulated and unstandardized ingredients and processes, illicit drugs vary in potency when compared to legal drugs such as nicotine, alcohol, or cannabis. Additionally, adolescents and young adults tend to overestimate the prevalence, amounts, and frequency with which their peers use drugs [35]. Uncertainties in the drug potency due to unregulated sources and inaccurate beliefs about peer drug use may contribute to risky drug use through accidental overdosing.

The dose domain also addresses the purity of the drug being used. As noted, illicit drugs often contain contaminants due to their unregulated manufacturing processes. As such, many fatal and non‐fatal overdoses result from additive ingredients of which the consumer was unaware [36]. In a recent study of current heroin users, being given information about the probability of impurities in a hypothetical sample of heroin, as well as the likelihood of a fatal overdose, impacted participants’ reported likelihood that they would use the sample [37]. As stated by the researchers, “These data provide empirical evidence that persons who use heroin might adjust their drug‐use behavior when they are provided with clear information, such as a positive result on a fentanyl test strip…” and the researchers were careful to emphasize that probability‐discounting was greater for overdose risk than for sample impurity, underscoring “the importance of targeted public health messaging that equates sample impurity with overdose likelihood” ([37], p. 225–226).

##### 2.1.5.1 Practical Application

Safety enhancement measures that can be taken in the dose domain may include identifying safety precautions during sourcing, the intended dosage before use, drug purity testing such as fentanyl test strips, drug replacement options, and desired effects, as well as psychoeducation on dosing, drug interactions, and exploration of myths about peer drug use.

#### 2.1.6 Domain 6: Administration

The administration domain of the DUSEM addresses how a drug enters a consumer's body. Routes of administration influence the onset, effects, duration, and safety of drug doses. Additionally, some methods of administration require drug preparation and delivery equipment such as smoking pipes, mugs/glasses, syringes, gauze, or vaporizers. The administration domain encapsulates safety measures throughout the preparation and ingestion of drugs.

Although the various administration methods continue to evolve, the overarching categories are enteral, parenteral, and others [38]. Each method of drug administration has benefits, disadvantages, and strategies consumers can use to enhance their drug‐use safety.

Enteral administration means that the drug is absorbed via the gastrointestinal tract, including oral, sublingual, and rectal administration of the drug. Enteral administration methods vary; however, benefits may include convenience, moderate absorption rates compared to smoking or injection, gradual onset, and decreased risk of infection or transmissible diseases. Disadvantages may include gradual onset leading to unintentional overdosing, irritation of the gastrointestinal tract, variable onset of effects, and less efficient than other methods such as injection or smoking [38].

Parenteral administration means that the drug is absorbed through routes other than the gastrointestinal tract. This includes injection methods such as intravenous, intramuscular, and subcutaneous. Advantages of parenteral administration include rapid onset, increased efficiency when compared to oral administration, and consumers have more control of dosage effects [38]. Disadvantages include increased risks of infections, rapid onsets and efficiency leading to overdoses, shorter effects when compared to oral administration, and requiring equipment such as syringes, water, and sterilization materials.

Other common administration methods include inhalation, such as smoking or vaporizing, and intranasal methods, such as nasal inhalers or snorting. These methods also bypass the gastrointestinal tract as drugs are absorbed through either the nasal mucosa or lung capillaries. Advantages of these methods include rapid onset, decreased risk of infection compared to injection, and lower dose than required for oral or injection. Disadvantages include difficulty with dose regulation, airway irritation, and may require expensive equipment for methods such as vaporization [39].

##### 2.1.6.1 Practical Applications

Practical applications within the administration domain might include education on administration and absorption methods, overdose signs and symptoms, sterilization techniques, safe preparation methods, and infection disease prevention protocols.

#### 2.1.7 Domain 7: Recovery

The recovery process for drug administration begins before the drug is administered and addresses recovery from acute and chronic drug‐use events. Because drugs vary in their effects, administration, absorption, metabolism, and elimination, education in this domain will require exploration of other domains such as knowledge, dose, and administration. Additionally, because individual drug consumers vary by physical and mental health issues, domains such as set and setting and motivation would also require consideration.

However, basic tenets of recovery from an acute drug‐use experience include adequate hydration and nutrition beforehand and during the drug‐use experience, followed by adequate nutrition, rest, sleep, and hydration following the event. Additionally, education about remedies, such as aspirin (acetylsalicylic acid) or ibuprofen for alcohol hangovers while avoiding acetaminophen, may aid in acute recovery from drug‐use experiences [30]. Acute recovery issues may include responses to overdoses and education about how to administer first aid or overdose prevention drugs such as naloxone.

Chronic recovery education may address the psychophysiological benefits and risks of ongoing use of various substances. To reiterate, the recovery domain will require individualized attention to other domains and their role in drug‐use experiences. For example, someone planning from the recovery domain may have varied benefits and side effects of chronic use of cannabis depending on the dose and the administration.

##### 2.1.7.1 Practical Applications

Applications from the recovery domain could include education about first aid, drug metabolism, administration of lifesaving drugs such as naloxone, overdose prevention methods, nutritional information, sleep tracking, and education about common psychophysiological risks and benefits of chronic and acute drug‐use experiences.

#### 2.1.8 Domain 8: Evaluation

The evaluation domain of the DUSEM is used to evaluate drug consumers’ relationships with drugs. In this domain, drug consumers are prompted to reflect on the benefits and side effects they have experienced based on their drug use. This can be used to evaluate chronic or acute drug‐use experiences and help determine future courses of action. This domain can employ skills and strategies based on motivational interviewing [8] in helping drug consumers determine if their drug use is causing any side effects, which would necessitate behavior changes within any domain of the DUSEM, such as changing dosage (dose), taking breaks (recovery), changing drugs (dose) or administration, or addressing underlying psychophysiological issues (motivation, set).

##### 2.1.8.1 Practical Applications

Applications from the evaluation model might include journaling, comparing actual intake to pre‐established goals, compiling pro/con lists of acute or chronic experiences, logging use events, and getting feedback from family/peers regarding their drug use.

### 2.2 Model Applications

The DUSEM provides an opportunity for revision of the attitudes and biases toward drug‐related behaviors and persons using drugs. Working with the populations engaging in drug‐related behaviors presents unique challenges for the providers. Allowing professionals to consider the political and cultural contexts for care provision is likely helpful. The DUSEM serves a dual purpose. First, it destigmatizes drug‐related behaviors by providing an alternative lens for contextualizing drug use. Second, DUSEM is a tangible tool for teaching novice mental health professionals using a bias‐reducing model to conceptualize drug use. It may also help create a cultural shift for seasoned practitioners, as the harm reduction philosophy still conveys a strong bias toward drug‐use reduction and cessation. Hence, the two main areas for application of DUSEM are helping professionals’ education (including continuing education) and clinical practice when collaborating with other healthcare professionals or for use in client psychoeducation.

Although this model aims to destigmatize drug use, negative attitudes about drugs and the people who use them are pervasive and embedded in many educational and healthcare settings [9]. Barriers to implementing harm reduction strategies such as the DUSEM often include resource and funding constraints, provider burnout, and communication with helping professionals, community members, and stakeholders who do not have a harm reduction orientation [40]. Managing and overcoming these barriers is integral to the success of harm reduction strategies and destigmatizing drug use. Helping professionals can use ongoing training, team‐based care, community engagement, and consumer and community education as methods of increasing awareness of the goals and methods of harm reduction and the DUSEM.

## 3 Limitations

The DUSEM is a conceptual educational model that has yet to be tested for its psychometric principles and efficacy. In that sense, this article should be considered a starting point rather than a final product. Although each domain is based on scientific peer‐reviewed studies and perspectives, integrating and applying this information requires further studies. Initial pilot studies in various settings might include small sample qualitative studies to explore helping professionals and their clients’ experiences utilizing the DUSEM. Qualitative studies could provide empirical evidence of this model's strengths, limitations, and target audiences. Research that deliberately incorporates the perspectives and experiences of people who use drugs is vital, given the historically limited inclusion of this community in programming and policy decisions that directly impact its constituents [41].

Additionally, as the DUSEM was designed to aid in destigmatizing drug use, drug use remains controversial. The secrecy and stigma about drug use may make the methods of delivery of this model imperative as audiences’ and consumers’ attitudes will vary. The model domains were developed to loosely convey longstanding concepts of drug use to serve as an educational guide; however, educators and drug consumers should exercise caution when educating from this model as its development is ongoing.

## 4 Future Research

Future research on the DUSEM will include qualitative studies to guide the application of the domains of this model. Additionally, qualitative studies could be used to help determine the efficacy and effects of activities used within this model. This model serves as an educational guidepost rather than a specific curriculum. Thus, qualitative studies could help determine activities to guide curriculum development and the efficacy of its application.

Future quantitative studies could be used to explore the factors within this model to determine their validity and uncover any additional factors that may contribute to drug‐use safety enhancement. Additionally, principal component analyses and other factor analyses can aid in developing questionnaires and interventions to assess drug‐use safety within each domain.

## 5 Conclusion

The DUSEM is an educational tool that is intended to increase the safety of drug consumers. This model was developed to be pragmatic and understandable to clinical professionals, researchers, and the public. The eight domains in this model can be used to increase awareness of various facets of drug‐use safety through education and the development of practical strategies and tools. Future studies will determine a core curriculum and develop interventions specific to target audiences.

## Author Contributions

The original concept for the DUSEM theoretical frame was created, structured, and drafted by Edward T. Dunbar Jr. Alison L. Brennan and Ania Bartkowiak engaged in revision and assisted in deepening the manuscript's intellectual scope. All authors participated in reviewing, editing, and approving the final version of this manuscript.

## Conflicts of Interest

The authors declare no conflicts of interest.

## Data Availability

This manuscript is a theoretical contribution, and a proof‐of‐concept study is needed in the future to validate its applicability and usefulness in the field. There was no data set the DUSEM theoretical framework is based on.
